# The predictive role of early recurrences of atrial arrhythmias after pulmonary vein cryoballoon ablation. Is blanking period an outdated concept? Insights from 12-month continuous cardiac monitoring

**DOI:** 10.1186/s12872-021-02300-2

**Published:** 2021-10-07

**Authors:** Karapet V. Davtyan, Arpi H. Topchyan, Hakob A. Brutyan, Elena N. Kalemberg, Maria S. Kharlap, Georgiy Yu. Simonyan, Andrey A. Kalemberg, Maria V. Kuznetsova

**Affiliations:** National Medical Research Center for Therapy and Preventive Medicine , Petroverigskiy Lane 10-3, Moscow, Russia 101990

## Abstract

**Background:**

Early recurrences of atrial arrhythmias (ERAA) after atrial fibrillation (AF) catheter ablation do not predict procedural failure. A well-demarcated homogeneous lesion delivered by cryoballoon is less arrhythmogenic, and the recommended three-months blanking period may not refer to cryoballoon ablation (CBA).

**Objective:**

We aimed to evaluate the predictive role of ERAA after second-generation CBA using an implantable loop recorder.

**Methods:**

This prospective observational study enrolled 100 patients (58 males, median age 58) with paroxysmal/persistent AF undergoing pulmonary vein (PV) CBA using second-generation cryoballoon with simultaneous ECG loop recorder implantation. The duration of follow-up was 12 months, with scheduled visits at 3, 6 and 12 months.

**Results:**

99 patients from 100 completed the 12-month follow-up period. ERAA occurred in 31.3 % of patients. 83.9 % of patients with ERAA also developed late recurrences. The 12-month freedom from AF in patients with ERAA was significantly lower than in those without ERAA (p < 0.0001). Non-paroxysmal AF and longer arrhythmia history were associated with increased risk of both early (HR 3.27; 95 % CI 1.32–8.08; p = 0.010 and HR 1.0147; 95 % CI 1.008–1.086; p = 0.015, respectively) and late recurrences (HR 3.89; 95 % CI 1.67–9.04; p = 0.002 and HR 1.0142; 95 % CI 1.007–1.078; p = 0.019, respectively) of AF. ERAA were another predictor for procedural failure (HR 15.2; 95 % CI (6.42–35.99; p = 0.019).

**Conclusions:**

ERAA occurred in the third of the patients after PV second-generation CBA and are strongly associated with procedural failure. Longer duration of AF history and persistent AF are independent predictors of AF’s early and late recurrence.

## Introduction

Catheter-based pulmonary vein isolation (PVI) is a well-established, effective and safe treatment option for drug-resistant paroxysmal and persistent atrial fibrillation (AF) [1]. The post-ablation three-month blanking period was recommended to blank the early atrial tachyarrhythmias presumably associated with post-ablation tissue edema, inflammation, and non-homogenous tissue lesion [2, 3]. In addition, it was traditionally considered that early recurrences of atrial arrhythmias (ERAA) do not predict long-term AF recurrence after PVI [4].

Pulmonary vein cryoballoon ablation (CBA) is a valuable alternative to radiofrequency ablation (RFA) [5]. The cryoballoon delivers a well-demarcated homogenous continuous lesion encircling PV antrum [6], which is less arrhythmogenic than the indistinct RFA-associated lesions [2, 7, 8]. Considering the discreet and focused fashion of lesion formation during cryoablation [8], which includes the consecutive phases of freezing/thawing, hemorrhage/inflammation, replacement fibrosis and apoptosis [9], we hypothesized that the recommended three-months blanking period did not refer to CBA, and the predictive role of ERAA after CBA remains unclear. However, data evaluating the predictive role of ERAA using implantable loop recorder (ILR) are limited to a small-sample single centre study 10]. Therefore, this study aimed to evaluate the significance of ERAA in predicting 12-month PV CBA failure in a larger sample of patients using an ILR.

## Methods

This prospective observational study (ClinicalTrials.gov NCT03587181, first posted date - July 16, 2018) consecutively enrolled 100 patients with symptomatic (≥ EHRA2b), drug-resistant persistent/paroxysmal AF, who underwent primary PV cryoballoon ablation (CBA) with simultaneous ECG loop recorder (ILR) (Reveal XT, Medtronic) implantation from April 2017 to October 2018 at National research center for therapy and preventive medicine (Moscow, Russia). We developed the study protocol in accordance with the Declaration of Helsinki, and the Independent Ethics Committee of the National medical research center for therapy and preventive medicine approved it. All patients signed the written informed consent before enrollment.

Inclusion criteria were age ≥ 18 years, drug-resistant (> 2 antiarrhythmic drugs (AADs)), symptomatic (≥ EHRA IIb class) paroxysmal/persistent AF. The exclusion criteria were significant structural heart disease (clinically significant coronary artery disease, valvular pathology, cardiomyopathies), left ventricular ejection fraction < 35 %, the history of previous catheter-based or surgical AF treatment, severe comorbidities (acute decompensation, injury or failure in other organ systems).

Patients discontinued all AADs at least two days before ablation. Amiodarone was stopped one month before the procedure. We followed the minimally interrupted oral anticoagulation therapy strategy before ablation: the last dose of apixaban and dabigatran was given the evening before ablation, and rivaroxaban was discontinued the morning one day prior. Warfarin was interrupted two-three days before the ablation to maintain the international normalized ratio range of 1.5–2.0.

Preprocedural transesophageal echocardiography was performed ≤ 24 h before ablation to exclude left atrial appendage thrombosis. PV CBA procedures were carried out under continuous intravenous sedation with propofol. First, intracardiac echocardiography guided (Acuson AcuNav 10-French, Biosense Webster) transseptal puncture was performed in the fossa ovalis using an 8 Fr. transseptal sheath (SL0, St. Jude Medical) with Brockenbrough needle. Intravenous heparin was administered at the time of transseptal puncture (target activated clotting time ≥ 250 s). Next, left atrial angiography was performed for left atrium (LA) and PV anatomy visualization. After that, the transseptal sheath was exchanged to cryoballoon delivery system FlexCath (Medtronic, USA, 12Fr), and second-generation cryoballoon Arctic Front Advance (28 mm balloon size, Medtronic, USA) with Achieve diagnostic catheter (Medtronic, USA) was advanced via the delivery system to the LA. Initially, we positioned Achieve catheter proximally at the PV ostium to map venous electrical activity and then inserted it distally into the PV to support cryoballoon positioning.

The freeze cycle of 180 s was started after obtaining a complete occlusion of the PV confirmed by dye injection. When possible, the time to PVI was monitored during freezing. We interrupted the application if PVI was not achieved < 90 s. Freezing was also interrupted if online PVI monitoring was unavailable and the dynamics of temperature drop was insufficient. We performed CBA in the clockwise direction (left superior PV, left inferior PV, right inferior PV, right superior PV). While performing CBA in the right PVs phrenic nerve was stimulated (30 bpm, 24mA), and diaphragmatic contraction by abdominal palpation was monitored. In the case of phrenic nerve palsy, we had immediately stopped ablation and had deflated the cryoballoon. After completing CBA, we remapped each PV to confirm the entrance and exit block in the PV antrum. If necessary, additional applications were performed to complete PVI.

The ILR (Reveal XT, Medtronic, USA) was implanted in the left parasternal area at the second intercostal space.

Oral anticoagulation therapy was restarted in 4 h after control echocardiography, excluding pericardial bleeding.

Patients with persistent AF continued AADs for three months after ablation. Patients with paroxysmal AF were discharged without any AADs.

The follow-up duration was 12 months, with scheduled visits in 3, 6 and 12 months. At each follow-up visit, the data regarding clinical events and healthcare utilization was collected. A 12-lead ECG was recorded, and data from the ILR were transmitted to the computer. Then two certified cardiac electrophysiologists performed a thorough analysis of retrieved ILR data.

Cardiac pauses > 3 s, bradycardia < 45 beats per min(bpm), ventricular tachycardia > 150 bpm, rapid ventricular tachycardia > 180 bpm, and AT/AF were the triggers to initiate the automatic ECG recording. In addition, patients could initiate ECG recording using an external activator device at the onset of the arrhythmia episode.

We defined ERAA as a clinical or subclinical ILR-detected AF/AT episodes with a duration of at least 2 min (due to ILR rhythm classification algorithm [11] analyzing RR variability over two minutes) occurring within post-ablation three months. AF recurrences occurring > 3 months after ablation were classified as PV CBA failure.

In cases of symptoms suggestive of AF recurrence, extra follow-up visits were performed.

Depending on early atrial arrhythmia recurrence (ERAA), patients were divided into patients with and without ERAA.

The ILR was removed after the completion of the study.

### Statistical analysis

Continuous variables were presented as mean ± standard deviation (SD) and median (Me) and interquartile range (IQR), categorical variables – as frequencies. Shapiro-Wilk test was conducted to evaluate the normality of the data. Comparisons between the two groups were performed with the Mann-Whitney U test, chi-square test with phi coefficient and 2-sided Fisher’s exact test, as appropriate. Kaplan-Meier analysis with the log-rank test was run to estimate the efficacy of PV CBA. Cox proportional hazard model was used to evaluate predictors for AF recurrence after CBA: the hazard ratio (HR) and 95 % confidence intervals (CI) were calculated per 1-unit of change in each continuous variable. A two-tailed p-value ≤ 0.05 was regarded to be significant. Statistical analysis was performed using Stata v15.0 for Windows (StataCorp., USA).

## Results

### Patients characteristics

We consecutively recruited one hundred patients with drug-resistant non-valvular AF who underwent primary PV CBA between April 2017 and October 2018. The patients’ median age was 58 (54–64); 58 (58 %) were males. Eighty-eight patients (88 %) had paroxysmal AF. The median duration of AF history was 4.5years. Concomitant arterial hypertension was found in more than two-thirds of the cases (71 %). Baseline characteristics of the study population are presented in Table 1.

**Table 1:** Baseline characteristics of the patients

Female n (%)	42 (42.4 %)
Age (years)	58 [54–64]
Age at the AF manifestation (years)	54 [46–61]
Paroxysmal AF n (%)	88 (88 %)
Duration of AF history (years)	4.0 [2–7]
BMI (kg/m^2^)	29.5 [27.5–32.3]
AH n (%)	71(71 %)
History of TIA/stroke n (%)	6(6 %)
DM n (%)	4(4 %)
CHA_2_DS_2_VASc score	2 [1–3]
LVEF (%)	63 [60–66]
LA size (mm)	41 [39–44]

Values are presented as median [25th–75th percentile] with interquartile range or n (%)

*AF* atrial fibrillation, *BMI* body mass index, *AH* arterial hypertension, *TIA* transient ischemic attack, *AAD* antiarrhythmic drug, *OAC* oral anticoagulant, *DM* diabetes mellitus,* LVEF* left ventricular ejection fraction, *LA* left atrium

### Procedural characteristics

Acute PV isolation was achieved in 99.5 % PV. In one patient, the right superior PV was not isolated due to phrenic nerve palsy. Median procedural and fluoroscopy time were 115 [105–135] and 24.3 (19.5–30.05) min, respectively. The median number of applications was 1 [1] per vein, with the median duration of freezing 220 [180–240] seconds with the median nadir temperature − 48 °C. Complete PV occlusion during freezing was obtained in 94.8 % of PV.

Procedure-related adverse events were observed in two patients. One patient developed a phrenic nerve palsy, which was resolved in two days, according to chest X-ray data. The other patient developed the ILR pocket site infection in two weeks, and the device was removed at the patient’s request.

### Clinical follow-up

Ninety-nine patients from 100 completed the 12-month follow-up period. One patient was excluded from the study due to ILR explantation on the patient’s request.

**Fig. 1:**
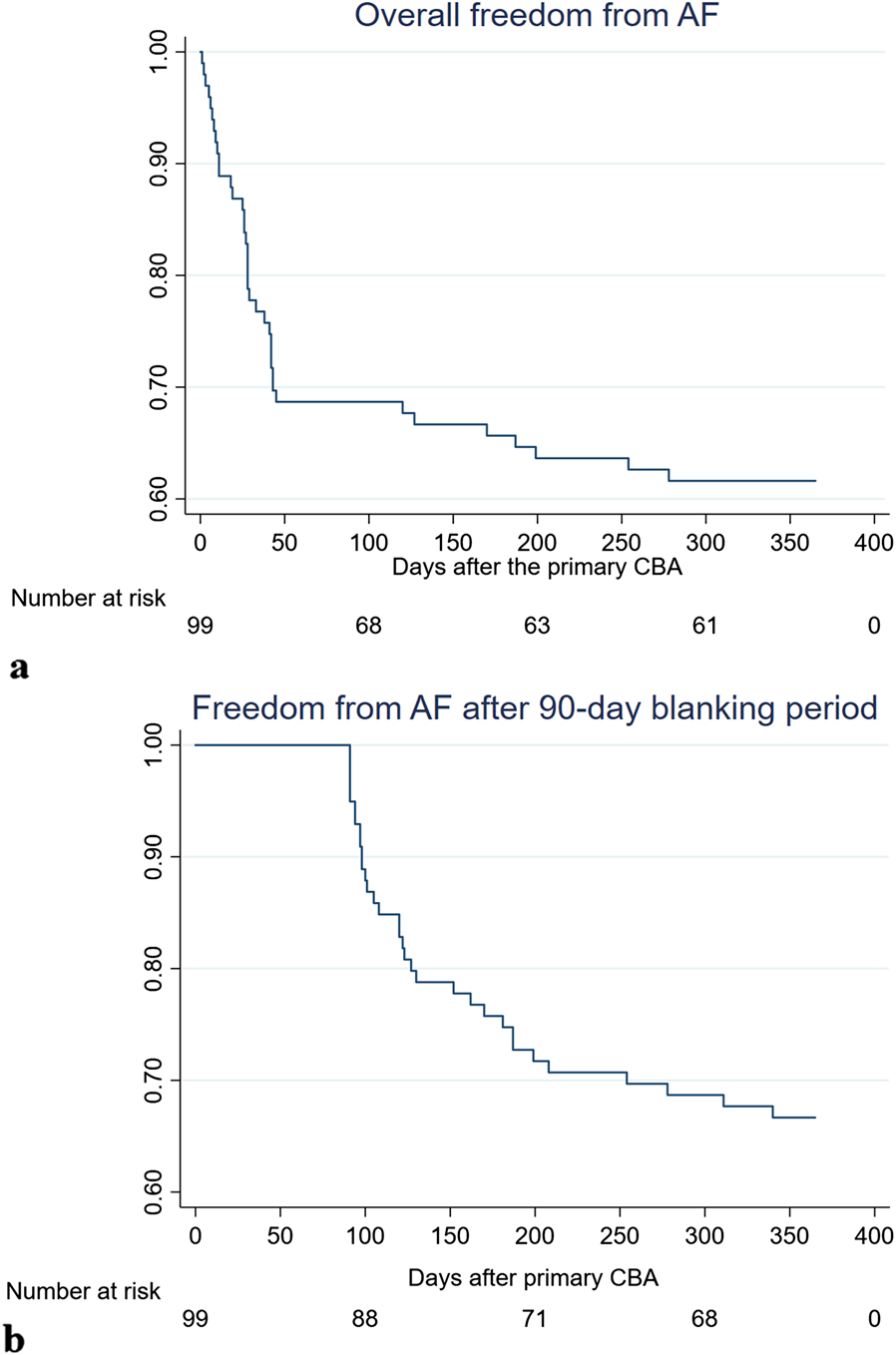
Kaplan-Meier curves of 12-month freedom from AF **a** without considering the blanking period, **B** considering the blanking period. *AF* atrial fibrillation, *CBA* cryoballoon ablation

The 12-month PV CBA success rate defined by continuous rhythm monitoring (without considering the blanking period) was 65.7 % (65/99) (Fig. 1a). The overall success rate considering the blanking period was 66.7 % (66/99) (Fig. 1b).

We observed a strong positive correlation between the ERAA rate detected by ILR and standard monitoring methods (resting ECG/24-hour Holter monitoring)—the phi coefficient was 0.724.

**Fig. 2:**
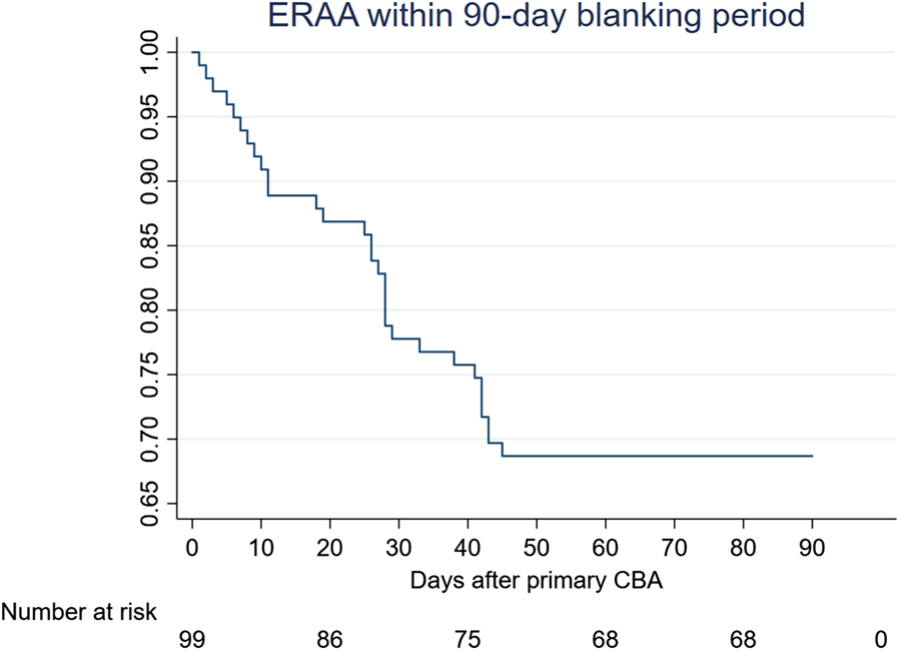
Time to the first recurrence of early atrial arrhythmias after the index pulmonary vein cryoballoon ablation. All cases of early recurrences occurred within 45 days after the procedure

ERAA occurred in 31.3 % (n = 31) patients; 30 patients from them (96.7 %) had AF recurrences, and one patient developed cavotricuspid atrial flutter (on the 41st post-ablation day). He had no subsequent arrhythmia after cavotricuspid isthmus ablation. In all patients, ERAA were observed within 45 days after ablation (Fig. 2).

**Fig. 3:**
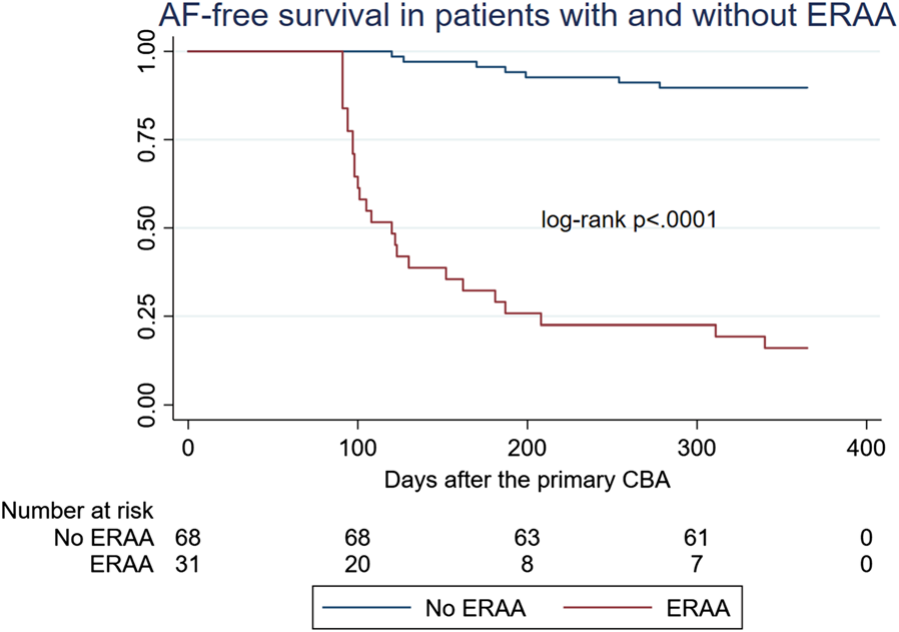
12-month freedom from atrial fibrillation in patients with and without early recurrences of atrial arrhythmias

The analysis of the time course of ERAA episodes revealed the following tendency: approximately 41.9 % of the patients (n = 13) had developed ERAA within 15 days, which was followed by a relative plateau phase for ten days; 58.1 % of the patients (n = 18) developed the first episode of ERAA in 25-45-th post-ablation days. We detected late AF recurrences beyond the 90-day blanking period in 83.9 % of patients with ERAA (26 from 31). AF-free survival of patients with early recurrences was significantly lower than those without ERAA (p < 0.0001) (Fig. 3).

The analysis of the four patients with early AF relapse without subsequent arrhythmia recurrences revealed the following: two patients developed brief clinically irrelevant AF episodes (with a duration of 20 and 8 min) in 19 and 43 days, respectively. In one patient, AF relapse occurred on the sixth post-ablation day and was associated with electrolyte imbalances due to dehydration, and in the other patient, AF recurred the next day after the ablation due to severe emotional distress.

Late recurrences of AF without prior ERAA occurred only in six patients (6.06 %).

Longer AF history and persistent AF were associated with increased risk of ERAA in univariate and multivariate Cox regression analysis (Table 2).

**Table 2:** Factors associated with ERAA in univariable and multivariable Cox regression analysis

Univariable analysis				Multivariable analysis			
	HR	95 % CI	p-value		HR	95 % CI	p-value
Age	1.012	0.974–1.052	0.533				
Sex	0.933	0.457–1.905	0.850				
Duration of AF history	1.041	1.004–1.080	0.029	Duration of AF history	1.047	1.008–1.086	0.015
AH	1.405	0.605–3.260	0.429				
Obesity	0.887	0.434–1.812	0.744				
PersAF	2.951	1.208–7.209	0.018	PersAF	3.272	1.325–8.082	0.010
LA size	1.028	0.933–1.131	0.578				
LVEF	1.002	0.948–1.060	0.932				

*AF* atrial fibrillation, *AH* arterial hypertension, *PersAF* persistent AF, *LA* left atrium, *LVEF* left ventricular ejection fraction, *HR* hazard ratio, *CI* confidence interval.

*Hazard ratio in continuous variables were calculated per 1-unit of change (1 year, 1 mm).

**Fig. 4:**
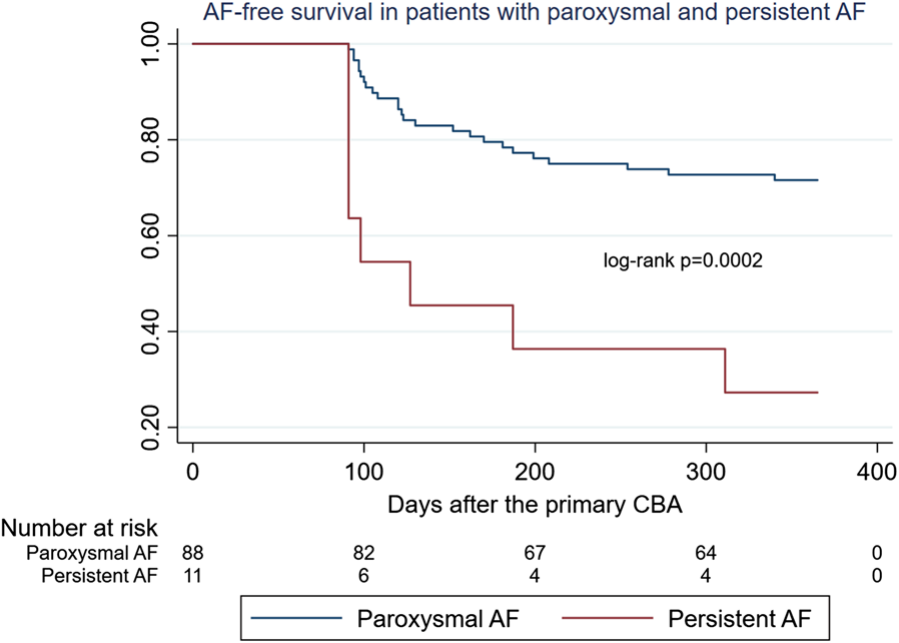
12-month freedom from atrial fibrillation in patients with paroxysmal and persistent atrial fibrillation

It is interesting to notice that persistent AF and longer duration of AF history were also significantly associated with 12-month PV CBA failure (Table 3). The 12-month freedom from AF was 71.6 % in patients with paroxysmal AF (25 from 88) and 27.3 % in patients with persistent AF(8 from 11) (p = 0.0002) (Fig. 4).

ERAA was another independent predictor associated with increased risk of late recurrences of AF after index PV CBA (HR 15.2, 95 % CI 6.42–35.99; p < 0.0001) (Table 3).

**Table 3:** Factors associated with late recurrences of AF after pulmonary vein cryoballoon ablation

Univariable analysis				Multivariable analysis			
	HR	95 % CI	p-value		HR	95 % CI	p-value
Age	1.018	0.979–1.059	0.363				
Sex	0.940	0.460–1.919	0.866				
Duration of AF history	1.059	1.020–1.099	0.002	Duration of AF history	1.042	1.007–1.078	0.019
AH	1.319	0.568–3.061	0.519				
Obesity	0.940	0.461–1.920	0.867				
PersAF	4.039	1.81–8.99	0.012	PersAF	3.886	1.671–9.036	0.002
LA size	1.024	0.929–1.128	0.635				
LVEF	1.007	0.949–1.068	0.820				
ERAA	17.421	7.443–40.777	< 0.0001	ERAA	15.20	6.418–35.996	0.0001

*AF* atrial fibrillation, *AH* arterial hypertension, *PersAF* persistent AF, *LA* left atrium, *LVEF* left ventricular ejection fraction, *ERAA* early recurrence of atrial arrhythmias, *HR* hazard ratio, *CI* onfidence interval

*Hazard ratio in continuous variables were calculated per 1-unit of change (1 year, 1 mm)

## Discussion

The post-ablation three month blanking period was recommended to blank the early atrial tachyarrhythmias presumably associated with post-ablation tissue edema, inflammation, and non-homogenous tissue lesion [3]. It is traditionally considered that ERAA does not predict long-term AF recurrence after PVI. On the other hand, RFA-associated tissue edema and inflammation are mainly resolved within 4 weeks [12], and the 90-day blanking period seems questionable. Clinical data also supported these findings [13, 14]: it has been shown that the likelihood of late AF recurrence increases with ERAA after the post-ablation first month [14]. Mature lesion formation by cryoablation lasts approximately four weeks and involves three distinct stages of histological changes: freeze/thaw phase (minutes to hours), hemorrhagic-inflammatory phase (days to a week), and replacement fibrosis phase (2–4 weeks) [9]. The well-demarcated homogenous continuous lesion around the PV antrum created by cryoballoon is less arrhythmogenic than the indistinct RFA-associated lesions [2]. All these data supported the hypothesis that the predictive role of ERAA after CBA remains unclear.

In our study, we detected ERAA in 32.2 % of patients. Furthermore, 83.9 % of these patients also developed late recurrences at 12-month follow-up. At first sight, different post-ablation antiarrhythmic management tactics may lead to discrepancies in ERAA development rates and, hence, further complicate the comparison with literature data. However, the study results conducted by Leong-Sit et al. reported [15] that although short-term use of AADs after AF ablation was associated with decreased ERAA rate, early AF recurrence on or off AADs during the initial 6-week blanking period is still a strong independent predictor of long-term AF recurrence.

Our results are highly comparable with the study data conducted by Pieragnoli et al. [10], evaluating the significance of ERAA on a smaller sample of patients with AF undergoing PV CBA. The ERAA rate was 28.3 % in continuously monitored 60 patients, and 82.3 % of them also had late recurrences [10]. Within-blanking AF relapse (HR 3.453; 95 % CI 1.544–7.722; p = 0.003) was independently associated with late AF recurrences. Non-paroxysmal AF (HR 3.113; 95 % CI 1.309–7.403; p = 0.010) and periprocedural AF (HR 2.309; 95 % CI 1.004–5.311; p = 0.049) were other predictors [10]. Our study determined the ERAA (HR 15.2; 95 % CI (6.42–35.99; p = 0.019), persistent AF type (HR 3.89; 95 % CI 1.67–9.04; p = 0.002) and longer duration of AF history (HR 1.0142; 95 % CI 1.007–1.078; p = 0.019) as independent predictors for late AF recurrence. Since persistent AF (HR 3.27; 95 % CI 1.32–8.08; p = 0.010) and duration of AF history (HR 1.047; 95 % CI 1.008–1.086, p = 0.015) were independent predictors for ERAA occurrence too, we may suggest the same underlying mechanism of early and late AF relapse. This is an important finding as many researchers consider that ERAA occurrence indicates a continuation of the preexisting initial AF mechanisms [16]. However, the short-therm prescription of AADs in the post-ablation 1–3 months might mask the ERAA occurrence and its’ association with the late recurrence. As we mentioned above, the rate of ERAA on AADs reported by Pieragnoli et al. [10] was lower (28.3 %) than in our study, predominantly off AADs (32.2 %). On the other hand, the rates of patients with true late recurrences in both studies were similarly high (82.3 % vs. 83.9 %). Thus, our data on a larger study sample confirmed the findings of the previous study [10] and have rationalized our hypothesis that the “blanking period” concept after PV CBA needs to be reviewed.

Mugnai et al. [17], evaluating the predictive role of ERAA on the incidence of late recurrences in patients with paroxysmal AF, who had undergone second-generation CBA, did not reveal any clinical factors associated with ERAA, and the blanking-period AF relapse was the only predictor of late AF recurrence. Another interesting finding of this study was the low rate of early arrhythmia recurrences − 8.76 %, which may be due to not using ILR; the authors monitored the cardiac rhythm using 24-hour Holter monitoring during follow-up visits, trans-telephonic monitors, or external loop recorders, which might compromise the accuracy of AF detection. The enrollment of patients with only paroxysmal AF is another explanation for within-blanking AF being the only predictor of PV CBA failure.

Another remarkable finding of our study is that all episodes of ERAA occurred within 45 days, and there was no pattern for patients between the timing of the ERAA and the rate of late recurrences. In contrast to our observation, Mugnai et al. [17] reported that ERAA were strongly associated with the definitive AT recurrence; particularly, all patients who had ERAA after 1.5 months from the ablation developed a late relapse. The authors concluded that these findings might indicate the necessity to shorten the “blanking period” following CBA to 1.5months. On the other hand, the propensity score-matched analysis of the clinical significance of early AF recurrences following CBA and RFA, performed by Tokuda et al. [18], revealed that early AF recurrences within 3–30 days and 31–90 days are independently associated with true AF recurrences for both CBA (p = 0.04 and p < 0.001, respectively) and RFA (p = 0.02 and p = 0.001, respectively). We suggest that such disparity in reported rates and the time course of ERAA occurrence and its significance on the incidence of late recurrences are due to the varying study designs, patients’ characteristics, and follow-up duration. Undoubtedly, further larger, multicenter studies are necessary to clarify these findings and answer the question of whether cryoballoon PVI procedure efficacy should be assessed with or without considering AF relapses within three post-ablation months.

## Limitations

There are some possible limitations to our study. First, this is a single-centre study, and the state-funded character of the trial predefined the study timeframe and the number of study participants. Second, the different AADs management strategies for patients with paroxysmal and persistent AF might also alter the results. Third, the small number of patients with persistent AF also limited its predictive significance. Fourth, we use the cut-off value of 2 min to define the AT/AF recurrence, limiting the comparison of study results with most literature data. Another limitation is that we did not use a remote monitoring system to improve patients’ data acquisition and assessment.

## Conclusions

ERAA occurred approximately in the third of the patients with paroxysmal and persistent AF after second-generation PV CBA within post-ablation 45 days. Longer duration of AF history and persistent AF are independent predictors of ERAA occurrence and late AF recurrence. More than 80 % of patients with ERAA after PV CBA also developed late AF recurrence. Considering our study results, we question the appropriateness of using the “blanking period” concept after cryoballoon ablation. However, further larger, multicenter studies are necessary to clarify these findings.

## Data Availability

The data that support the findings of this study are available from the corresponding author upon reasonable request.

## References

[1] Hindricks G, Potpara P, Dagres N, Arbelo E, Bax JJ, Blomström-Lundqvist C (2020). 2020 ESC Guidelines for the diagnosis and management of atrial fibrillation developed in collaboration with the European Association for Cardio-Thoracic Surgery (EACTS). Eur Heart J.

[2] Khairy P, Chauvet P, Lehmann J, Lambert J, Macle L, Tanguay J-F (2003). Lower incidence of thrombus formation with cryoenergy versus radiofrequency catheter ablation. Circulation.

[3] Richter B, Gwechenberger M, Socas A, Zorn G, Albinni S, Marx M (2012). Markers of oxidative stress after ablation of atrial fibrillation are associated with inflammation, delivered radiofrequency energy and early recurrence of atrial fibrillation. Clin Res Cardiol.

[4] Calkins H, Hindricks G, Cappato R, Kim Y-H, Saad EB, Aguinaga L (2018). 2017 HRS/EHRA/ECAS/APHRS/SOLAECE expert consensus statement on catheter and surgical ablation of atrial fibrillation. Europace.

[5] Kuck K-H, Brugada J, Fürnkranz A, Metzner A, Ouyang F, Chun KRJ (2016). Cryoballoon or radiofrequency ablation for paroxysmal atrial fibrillation. N Engl J Med.

[6] Kurose J, Kiuchi K, Fukuzawa K, Mori S, Ichibori H, Konishi H (2018). The lesion characteristics assessed by LGE-MRI after the cryoballoon ablation and conventional radiofrequency ablation. J Arrhythmia.

[7] Andrade JG, Dubuc M, Guerra PG, Macle L, Mondésert B, Rivard L (2012). The biophysics and biomechanics of cryoballoon ablation. PACE - Pacing Clin Electrophysiol.

[8] Khairy P, Dubuc M (2008). Transcatheter cryoablation part I: Preclinical experience. PACE - Pacing Clin Electrophysiol.

[9] Lustgarten DL, Keane D, Ruskin J (1999). Cryothermal ablation: Mechanism of tissue injury and current experience in the treatment of tachyarrhythmias. Prog Cardiovasc Dis.

[10] Pieragnoli P, Paoletti Perini A, Ricciardi G, Checchi L, Giomi A, Muraca I (2017). Recurrences in the Blanking Period and 12-Month Success Rate by Continuous Cardiac Monitoring After Cryoablation of Paroxysmal and Non-Paroxysmal Atrial Fibrillation. J Cardiovasc Electrophysiol.

[11] Hindricks G, Pokushalov E, Urban L, Taborsky M, Kuck KH, Lebedev D (2010). XPECT Trial Investigators. Performance of a new leadless implantable cardiac monitor in detecting and quantifying atrial fibrillation: Results of the XPECT trial. Circ Arrhythm Electrophysiol.

[12] Okada T, Yamada T, Murakami Y, Yoshida N, Ninomiya Y, Shimizu T et al. Prevalence and severity of left atrial edema detected by electron beam tomography early after pulmonary vein ablation. J Am Coll Cardiol. 2007;49(13):1436-42. 10.1016/j.jacc.2006.10.076. 10.1016/j.jacc.2006.10.076 17397672

[13] Joshi S, Choi AD, Kamath GS, Raiszadeh F, Marrero D, Badheka A (2009). Prevalence, predictors, and prognosis of atrial fibrillation early after pulmonary vein isolation: findings from 3 months of continuous automatic ECG loop recordings. J Cardiovasc Electrophysiol..

[14] Alipour P, Azizi Z, Pirbaglou M, Ritvo P, Pantano A, Verma A, Khaykin Y (2017). Defining blanking period post-pulmonary vein antrum isolation. JACC Clin Electrophysiol..

[15] Leong-Sit P, Roux JF, Zado E (2011). Antiarrhythmics after ablation of atrial fibrillation (5A study) six-month follow-up study. Circ Arrhythmia Electrophysiol.

[16] Gottlieb LA, Dekker LRC, Coronel R (2021). State-of-the-art review: the blinding period following ablation therapy for atrial fibrillation proarrhythmic and antiarrhythmic pathophysiological mechanisms. JACC Clin Electrophysiol.

[17] Mugnai G, de Asmundis C, Hünük B, Ströker E, Velagic V, Moran D (2016). Second-generation cryoballoon ablation for paroxysmal atrial fibrillation: Predictive role of atrial arrhythmias occurring in the blanking period on the incidence of late recurrences. Heart Rhythm..

[18] Tokuda M, Yamashita S, Matsuo S, Kato M, Sato H, Oseto H (2019). Clinical significance of early recurrence of atrial fibrillation after cryoballoon vs. radiofrequency ablation-A propensity score matched analysis. PLoS One..

